# The possible function of Flp1 in homologous recombination repair in *Saccharomyces cerevisiae*

**DOI:** 10.3934/genet.2018.2.161

**Published:** 2018-04-03

**Authors:** Huong Thi Thu Phung, Hoa Luong Hieu Nguyen, Dung Hoang Nguyen

**Affiliations:** NTT Hi-Tech Institute, Nguyen Tat Thanh University, Ho Chi Minh city, Vietnam

**Keywords:** Flp1, genetic screening, homologous recombination repair, Mus81, Sgs1

## Abstract

*Saccharomyces cerevisiae* Mus81 is a structure-selective endonuclease which constitutes an alternative pathway in parallel with the helicase-topoisomerase Sgs1-Top3-Rmi1 complex to resolve a number of DNA intermediates during DNA replication, repair, and homologous recombination. Previously, it was showed that the N-terminal region of Mus81 was required for its *in vivo* function in a redundant manner with Sgs1; *mus81_Δ120N_* mutant that lacks the first 120 amino acid residues at the N-terminus exhibited synthetic lethality in combination with the loss of *SGS1*. In this study, the physiologically important role of the N-terminal region of Mus81 in processing toxic intermediates was further investigated. We examined the cellular defect of *sgs1Δmus81_Δ100N_* cells and observed that although viable, the cells became very sensitive to DNA damaging agents. A single-copy suppressor screening to seek for a factor(s) that could rescue the drug sensitivity of *sgs1Δmus81_Δ100N_* cells was performed and revealed that Flp1, a site-specific recombinase 1 encoded on the 2-micron plasmid was a suppressor. Moreover, Flp1 overexpression could partially suppress the drug sensitivity of *mus81Δ* cells at 37 °C. Our findings suggest a possible function of Flp1 in coordination with Mus81 and Sgs1 to jointly resolve the branched-DNA structures generated in cells attempting to repair DNA damages.

## 1. Introduction

Mus81, a highly conserved DNA structure–selective endonuclease, is related to the XPF/Rad1 family of proteins involved in DNA nucleotide excision repair. Mus81 functions as a heterodimeric protein complex with a partner, namely Eme1 and Eme2 in humans, Eme1 in fission yeast, and Mms4 in budding yeast [1]–[3]. Its partner proteins are indispensable for stability and the nuclease activity of the complex [4].

The *mus81* mutants are hypersensitive to different types of DNA damaging agents including ultra violet irradiation, methyl methanesulfonate (MMS), hydroxide urea (HU), 2-phenyl-3-nitroso-imidazo [1,2-α] pyrimidine, cisplatin, doxorubicin, tirapazamine, and camptothecin [5]–[7]. Mus81 can cleave a numerous of branched-DNA structures that may form *in vivo* during many DNA transactions such as nicked Holliday Junctions (HJs), D-loop, replication forks with the lagging strand at the junction point, and 3′-flap [1],[3],[8]. Despite the fact that Mus81 was reported as a HJs resolvase because the affinity-purified Mus81–Eme1 complex exhibited marked activity on intact HJs [9]–[11], intact HJs are considered as generally poor substrates for this nuclease [1],[3],[8],[12]–[14]. Later study proved that the recombinant fission or budding yeast Mus81 complexes exhibited strong activity on intact HJs based on the tetramer formation [15]. In contrast, more recent work demonstrated that *Saccharomyces cerevisiae* Mus81–Mms4 functions as a single heterodimer in recombinational DNA repair and hardly cleaves intact HJs [16]. Interestingly, the activity of Mus81–Mms4 is cell-cycle regulated by the phosphorylation of Mms4 by Cdc28 (CDK) and Cdc5 (Polo-like kinase) which enhance the activity of Mus81 complex on intact HJs [17],[18]. Recently, a third cell cycle kinase, Cdc7-Dbf4 (DDK) was shown to be able to phosphorylate Mus81-Mms4 in conjunction with Cdc5 in an interdependent manner and this phosphorylation is also strictly required for Mus81 activation in mitosis [19]. Besides, Mus81-Mms4 was proved to relocalize to subnuclear foci with Rad1-Rad10 and Slx1-Slx4 in response to DNA damage and the relocalization correlated with the function of the complex endonuclease [20].

*S. cerevisiae* *MUS81* and *MMS4* genes were both identified in a synthetic lethality screen of *sgs1*Δ mutants [21]. Sgs1, a member of the ubiquitous RecQ family of DNA helicases was shown to form a stable complex with Top3 and Rmi1 which enhances the enzymatic activity of Sgs1-Top3 complex. Inactivation of constituents of Sgs1–Top3–Rmi1 complex resulted in the increased level of mitotic recombination, sister chromatid or chromosome exchanges, and genome instability, indicating that the complex plays critical role in homologous recombination repair pathway [22]. Importantly, the synthetic lethality of double deletion of *mus81* or *mms4* together with *sgs1* can be rescued by further deletion of recombination proteins, such as Rad51 or Rad52 [1],[8],[21],[23]. These results prove that Mus81 complex functions downstream of homologous recombination, being significantly involved in processing recombination intermediates in parallel or redundantly with Sgs1 complex [6],[8],[13],[23],[24].

Recently, our previous study showed the genetic and functional interaction of Rad27, an important nuclease involving in Okazaki fragment processing and base excision repair, and the Mus81 complex [25],[26]. The presence of Rad27 stimulated endonuclease activity of Mus81-Mms4 *in vitro* and vice versa, implicating the joint function of both enzymes in DNA replication or/and recombination. Besides, Srs2, a DNA helicase and DNA-dependent ATPase which is involved in DNA repair and checkpoint recovery, has been currently demonstrated to be able to promote Mus81–Mms4-mediated resolution of recombination intermediates [27]. Also, more recently, several enzymes were shown to be able to enhance the enzymatic activity of Mus81-Mms4 complex, such as heteropentameric replication factor C (RFC) complex and Esc2, an adaptor protein containing no known enzymatic domains, indicating the cooperation between Mus81 and these partners in the process of intermediates [28],[29]. The functional interaction of Mus81 complex and its partners depended on their physical interaction, specifically requiring the N-terminal region of Mus81 [26],[27]. Moreover, these physical and functional interactions are significantly important for cellular function of Mus81 in both budding yeast [26],[27],[30] and human [31]. Taking together all, Mus81–Mms4 is most likely to work with other proteins involved in a variety of DNA metabolisms including DNA repair, replication fork stability, and joint molecule formation/resolution during homologous recombination in order to safeguard the genome integrity.

Here, we further investigated the significance role of Mus81 N-terminus *in vivo* by performing a single-copy suppressor screening to seek for a factor(s) that can suppress the cellular defect caused by function loss of Mus81 N-terminal region. Unlike the mus81*_Δ120N_* mutant allele which is synthetic lethality when combined with *sgs1Δ*, the *sgs1Δmus81_Δ100N_* double mutant cells could survive, however, exhibited excessive sensitivity to DNA damaging agents. Through screening, we successfully recovered a candidate that can rescue the HU sensitivity of *sgs1Δmus81_Δ100N_* mutant cells, namely *FLP1*, a site-specific recombinase 1 which is encoded on the 2-micron plasmid. Accordingly, Flp1 is the possible suppressor of cellular defect caused by the dysfunction of Sgs1 and Mus81 when cells are challenged by DNA damages.

## 2. Materials and methods

### 2.1. Yeast strains

*S. cerevisiae* NJY1777 (*MATa ade2-1 ade3::hisG* ura3*-1 his3-11,15 trp1-1 leu2-3,112 lys2 mus81-10::KAN sgs1-20::hphMX4 can1-100 + pJM500-URA3-SGS1*) was a courtesy from Dr. Miki Ii at University of Alaska Anchorage (AK, USA) [32].

### 2.2. Drop dilution assay

The plasmid pRS325, a yeast episomal vector with a *LEU2* marker, harboring wild-type *MUS81* or *mus81* mutant alleles driven by *ADH1* promoter was transformed into NJY1777. The transformants were grown on plates containing selective synthetic defined media and a single colony from each of the transformants was inoculated into liquid media (1 mL) until saturation. Cell densities were adjusted to OD_600_ = 1 (∼2 × 10^7^ cells/mL) by diluting with dH_2_O, followed by spotting of 10-fold serial dilutions onto selective media plates containing with or without DNA damaging agents. The plates were incubated for 4 days at 30 °C. The complementation of *sgs1Δmus81Δ* synthetic lethality by *mus81* mutant alleles was performed in the presence of 5-FOA (uracil analog, 5-fluoroorotic acid). To test the drug sensitivity of *mus81* mutant alleles in the *sgs1Δ* null strain, cells which grew in the presence of 5-FOA were spotted onto plates containing indicated concentrations of DNA damaging agents.

### 2.3. Screening single-copy suppressors of sgs1Δmus81_Δ100N_ mutant

Yeast genomic DNA library was constructed by Sau3AI-partial digestion of genomic DNA of *S. cerevisiae* YPH499 strain (*MAT*a *ura3-52 lys2-801 ade2-101 trp1-Δ63 his3-Δ200 leu2-Δ1*). The fragmented genomic DNA with estimately 5.6 kb in length on average was ligated into BamHI-digested pRS315 plasmid, a yeast centromere vector with a *LEU2* marker. Ligation product was then transformed into *Escherichia coli* competent cells and the library plasmids were extracted and stored at −80 °C for long-term usage. Before screening, the yeast genomic DNA library was amplified using Plasmid Maxi Kit (QIAGEN, Hilden, Germany).

NJY1777 cell containing a plasmid harboring wild-type *SGS1* gene with a *URA3* marker was transformed with *mus81_Δ100N_* gene consisted in pRS314 plasmid, a yeast centromere vector with a *TRP1* marker. Transformants were grown in the selective media and transferred onto plates containing 5-FOA, producing double mutant *sgs1Δmus81_Δ100N_* cells. The double mutant cells were then transformed with yeast genomic DNA library. Transformants were grown in selective media for 24 hours at 30 °C, followed by replica plating onto the same medium supplemented with 20 mM HU. The plates were incubated for additional 3–4 days. Selected colonies that grew on HU plates were examined for HU resistant capability by drop dilution assay. The HU-resistant colonies were transferred to liquid medium, and total plasmids were isolated. To confirm single-copy suppression, recovered plasmids were retransformed into the *sgs1Δmus81_Δ100N_* mutant cells and examined for their ability to support cell growth in the presence of HU. Double-checked plasmids were analyzed by sequencing to identify genomic DNA fragments inserted. One of the analyzed plasmids contained the full length of *FLP1* gene.

## 3. Results

### 3.1. The significance of the N-terminal region of Mus81

**Figure 1.**
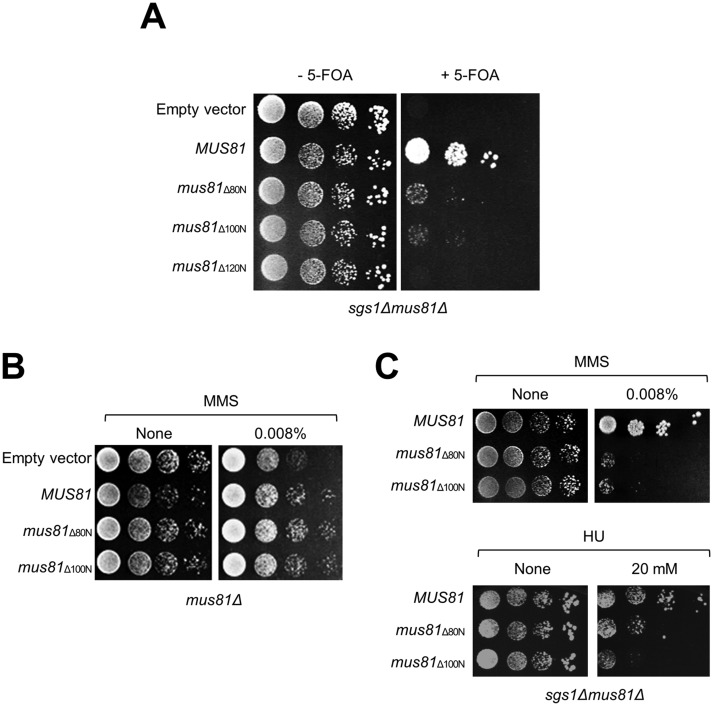
The cellular defect of *mus81_Δ80N_* and *mus81_Δ100N_*. (A) The complementation of *sgs1Δmus81Δ* synthetic lethality by *mus81* mutant alleles. (B) The MMS sensitivity of *mus81_Δ80N_* and *mus81_Δ100N_*. (C) The MMS sensitivity of *sgs1Δmus81_Δ80N_* and *sgs1Δmus81_Δ100N_*. The cells that grew in the presence of 5-FOA (in panel A) were spotted onto without or with indicated amount of MMS or HU concentration.

We previously found that the *sgs1Δmus81_Δ120N_* double mutant cell was synthetic lethality similarly to the *sgs1Δmus81Δ* double mutant, indicating that the deletion of the first 120 amino acids at the N-terminal region of Mus81 affected the cellular function of this enzyme ([26]; Figure 1A). In order to further define *in vivo* defects associated with the loss of Mus81 N-terminus, we examined the viability of *sgs1Δmus81_Δ80N_* and *sgs1Δmus81_Δ100N_* mutant cells using *sgs1Δmus81Δ* double mutant cells that were rendered viable by the presence of a plasmid expressing wild-type Sgs1. With the control empty vector transformed, in the presence of 5-FOA the *sgs1Δmus81Δ* cells had lost the plasmid containing *SGS1* and became lethal (Figure 1A). Expectedly, these cells developed normally in the presence of episomal copies of wild-type *MUS81* (Figure 1A). In contrast to the synthetic lethality of *sgs1Δmus81_Δ120N_*, we found that the presence of *mus81_Δ80N_* and *mus81_Δ100N_* alleles, despite of poor development, succeeded to support the growth of *sgs1Δmus81Δ* cells (Figure 1A). These findings show that a region between the 100^th^ and 120^th^ amino acid residues at the N-terminus of Mus81 is crucial for the function of Mus81 *in vivo* which is relevant to the cell survival in the absence of Sgs1. Here, it was observed that the deficient growth of the *sgs1Δmus81_Δ100N_* and *sgs1Δmus81_Δ80N_* double mutants represented the “bottleneck effect” where a size of population reduced sharply due to a sudden change of environment.

Next, MMS sensitivity of the *mus81_Δ80N_* and *mus81_Δ100N_* single mutant cells and the *sgs1Δmus81_Δ80N_* and *sgs1Δmus81_Δ100N_* double mutant cells were investigated. When wild-type *MUS81* was introduced into the *mus81Δ* deletion cells, they grew normally in the presence of MMS (Figure 1B). Unlike drug sensitivity phenotype of *mus81Δ* and *mus81_Δ120N_* [26], the *mus81_Δ80N_* and *mus81_Δ100N_* cells behaved like wild-type (Figure 1B). However, the *sgs1Δmus81_Δ80N_* and *sgs1Δmus81_Δ100N_* double mutants were sensitive to MMS and HU treatment (Figure 1C), indicating that the defect causing by the absence of the first 80 and 100 amino acid residues at Mus81 N-terminus, respectively, induced severe troubles to the cell, especially in dealing with DNA-lesion induced by DNA damaging agents. Overall, these results further demonstrated the critical function of Mus81 N-terminal region in homologous recombination repair pathways when cells are faced with accumulated DNA lesions caused by DNA damaging agents.

### 3.2. The single-copy suppressor screening to find out a factor(s) that can suppress the cellular defect causing by the dysfunction of N-terminal region of Mus81

We aim to seek for an alternative pathway that can cope with the loss of function of the important Mus81 N-terminal region. The results in Figure 1A point out that the region between the 100^th^ to 120^th^ amino acid residues at the N-terminus of Mus81 is indispensible for the cellular function of Mus81 in the absence of Sgs1 because removal of this small portion in the absence of Sgs1 led to cell death unavoidably. To perform the single-copy suppressor screening to define a suppressor of Mus81 lacking N-terminus mutant, we decided to maintain this small region due to its essential function *in vivo*. Thus, we chose the defective phenotype of *sgs1Δmus81_Δ100N_* cells (Figure 1C) to carry out the suppressor screening in order to identify a factor that can rescue this defect.

**Figure 2.**
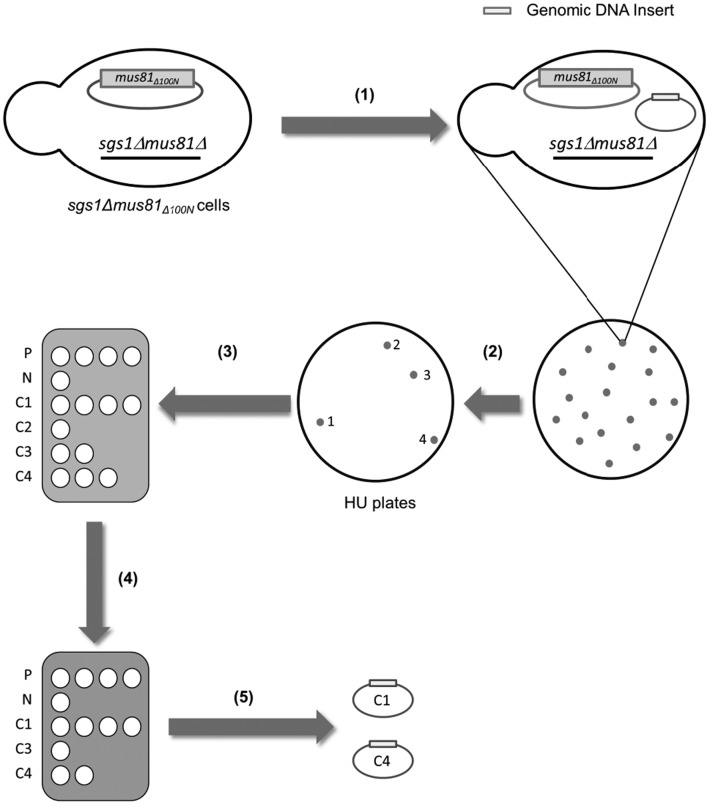
Scheme of the single-copy suppressor screening of drug sensitivity of the *sgs1Δmus81_Δ100N_* cells. (1) transformation with yeast genomic library, (2) replica onto HU plates, (3) and (4) drop dilution assay, (5) sequencing. Abbreviations: P, positive control; N, negative control; C1–4, representative colonies number 1 to 4.

As shown in Figure 2, the yeast genomic DNA library was transformed into the *sgs1Δmus81_Δ100N_* cells and the transformants were replicated onto plates containing 20 mM HU. Survival colonies were selected and serial-diluted spotted onto HU-plates in order to evaluate the HU resistant ability. Well growing cells were selected and the transformed plasmids were extracted. The plasmids were next transformed back into the *sgs1Δmus81_Δ100N_* cells and transformants were re-examined for HU-resistant capability. Finally, the plasmids producing resistant transformants were sequenced to define the possible genes involving in suppression of the Mus81_Δ100N_ dysfunction.

**Figure 3.**
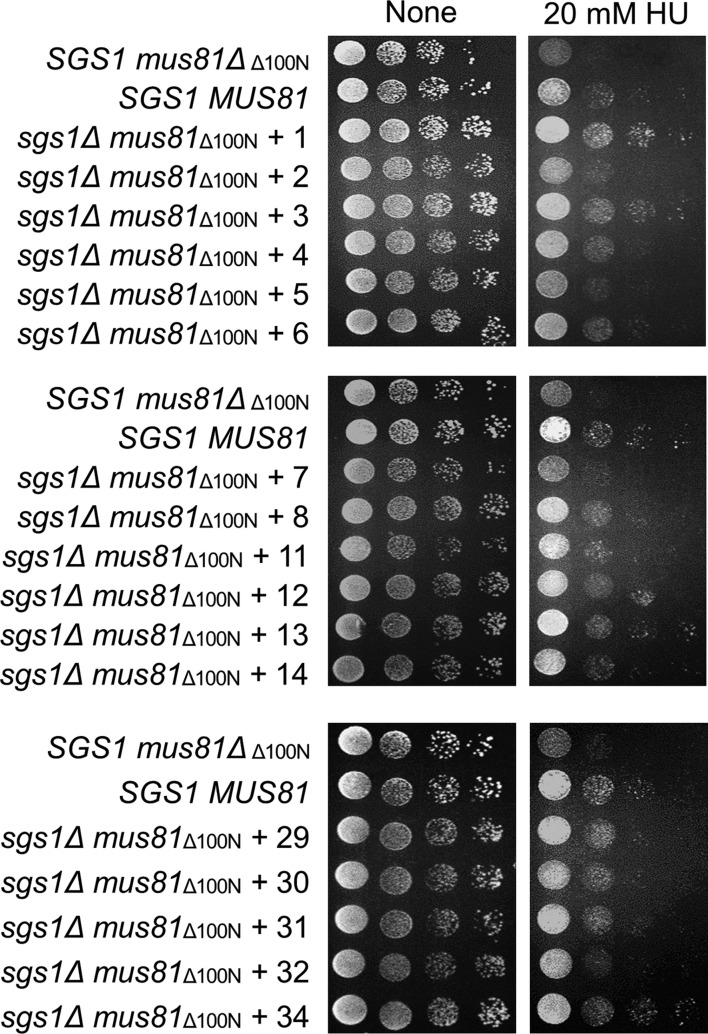
A drop dilution assay examining the transformant colonies that survive in the presence of HU. The *sgs1Δmus81_Δ100N_* cells containing yeast genomic DNA fragment which survived on HU plates after replica step were selected and serial-diluted spotted onto plates without or with 20 mM HU.

**Figure 4.**
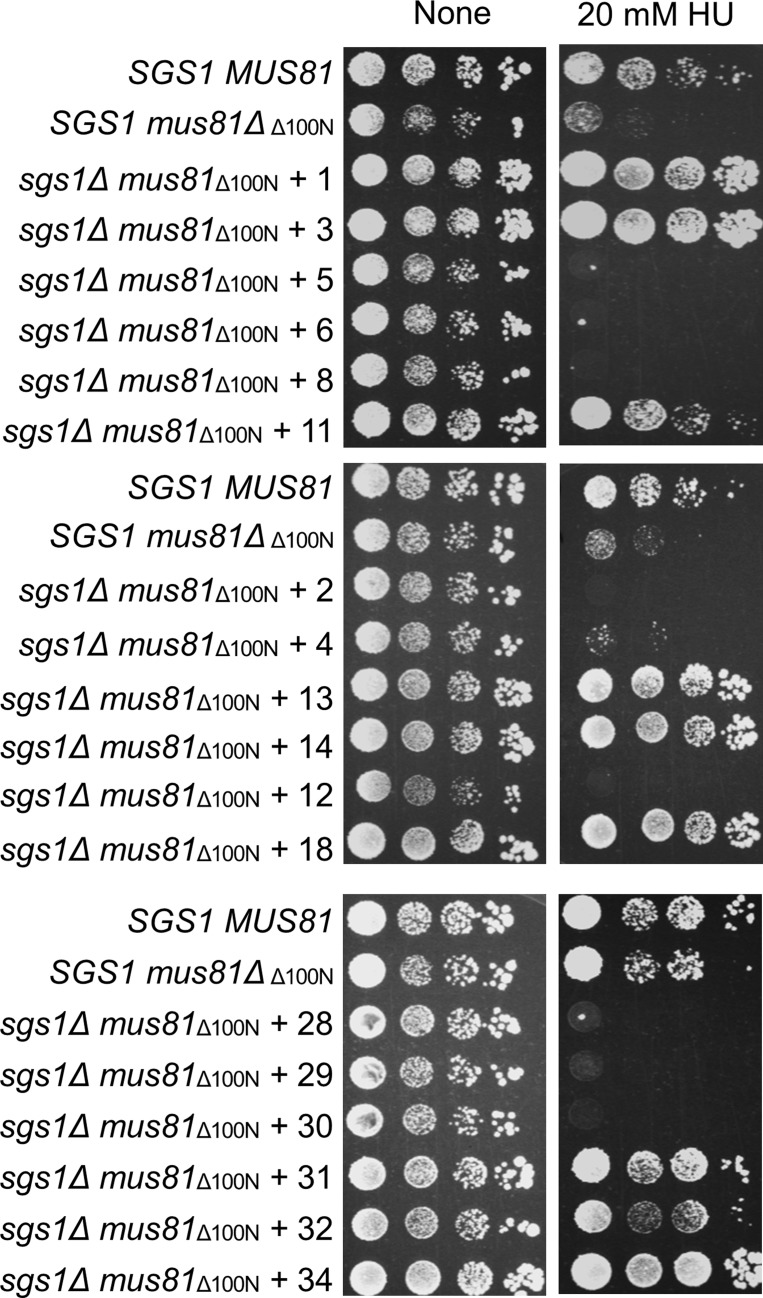
A drop dilution assay to confirm the suppression ability of the candidates. The *sgs1Δmus81_Δ100N_* cells were transformed with extracting plasmids from selected candidates and then serial-diluted spotted onto plates without or with 20 mM HU.

Collectively, after replica plating step, there were fifty-seven colonies that could grow on HU plates. Choosing those colonies and using drop dilution assay, we were able to examine the HU-resistant ability of fifty-four colonies (Figure 3). Among fifty-four checked colonies, forty-one were capable of suppress HU sensitivity of the *sgs1Δmus81_Δ100N_* mutant. There were twenty-three strong suppressors in comparison to wild-type cells (Figure 3, Table 1). Next, plasmids from forty-one colonies were extracted and re-transformed into the *sgs1Δmus81_Δ100N_* cells. Among forty-one candidate plasmids extracted, thirty-eight successfully created transformants. Then transformants were serial-diluted spotted onto plates containing HU to evaluate their survival. Among thirty-eight obtained transformants, only sixteen were capable of resisting to HU treatment (Figure 4, Table 1).

**Table 1.** Summary of the single-copy suppressor screening of *sgs1Δmus81_Δ100N_* mutant.

Candidate	Suppression	Confirmed Suppression	Gene sequence
1	+++	+++	*SGS1*
2	+	-	
3	+++	+++	*SGS1*
4	+	-	
5	+	-	
6	++	-	
7	-	/	
8	+	-	
9	NA	/	
10	NA	/	
11	+	++	NA
12	+	-	
13	++	+++	*SGS1*
14	+	+++	*SGS1*
15	-	/	
16	NA	/	
17	-	/	
18	+++	+++	*SGS1*
19	+	-	
20	-	/	
21	+++	+++	*SGS1*
22	-	/	
23	+++	+++	*SGS1*
24	+++	+++	*SGS1*
25	+	+++	*SGS1*
26	+	-	
27	++	+++	*SGS1*
28	+	-	
29	+	-	
30	+	-	
31	+	++	*FLP1*
32	+	-	
33	++++	NA	
34	+++	+++	*SGS1*
35	+	-	
36	F	/	
37	+++	+++	*SGS1*
38	+	-	
39	+++	+++	*SGS1*
40	+++		+++
41	-	/	
42	-	/	
43	-	/	
44	+++	NA	
45	++++	-	
46	++++	/	
47	++	-	
48	++++	-	
49	-	/	
50	+	-	
51	++++	-	
52	+++	-	
53	+++	-	
54	-	/	
55	++++	-	
56	-	/	
57	+	-	
Sum	41	16	

(+) suppressed; (+++) strong suppressed in comparison to positive control; (-) not suppressed.

_F_False positive; _NA_Not available

### 3.3. Flp1, a site-specific recombinase 1 encoded on the 2-micron plasmid is a suppressor of mus81 mutant lacking the N-terminal region

Sixteen plasmids that transformants created were able to grow well in the presence of HU were sequenced to identify the genomic DNA fragments inserted. Finally, it revealed fourteen plasmids harboring *SGS1* sequence including either or both of its upstream and downstream sequence and nearby region on chromosome XIII, jointly forming inserted fragment of approximately 6 kb. The presence of *SGS1* in screening results served as a positive control for our suppressor screening approach. The plasmid number 31 contained an upstream sequence (around 250 base pairs) and full length of a gene called *FLP1*, a site-specific recombinase 1 encoded on the 2-micron plasmid which is a multi-copy selfish extrachromosomal DNA element found in the nucleus in budding yeast. The pRS315 plasmid that we used to construct genomic library is the yeast centromere vector that does not harbor the 2-micron origin of replication and is maintained at a low copy number. Moreover, even yeast episomal plasmids do not contain the full-length Flp1 sequence and its upstream region, indicating that the presence of Flp1 sequence in screening result is definitely not derived from the used plasmid. Here, it is clear that the construct of genomic DNA fragment found in plasmid number 31 could guarantee the expression of functional Flp1 due to the presence of its native promoter in upstream sequence and completed coding sequence.

The 2-micron plasmid is relatively small (approximately 6.3 kb), and resides in most common strains of *S. cerevisiae* at steady-state copy number of 40–60 molecules per haploid cell [33],[34]. The plasmid harbors an origin of bidirectional DNA replication called ARS (autonomous replication sequence), *FLP1* gene, three other genes encoding proteins required for regulation of Flp1 expression, namely *REP1*, *REP2*, and *FAF1*, a set of small direct repeats forming a partitioning locus named STB, and two 599-bp inverted repeat sequences called Flp Recombination Targets (FRTs) [33],[35]–[38]. Flp1 with a couple of FRT sites residing in the plasmid in a head-to-head orientation belong to the amplification system which is responsible for the 2-micron plasmid self-replication [38].

**Figure 5.**
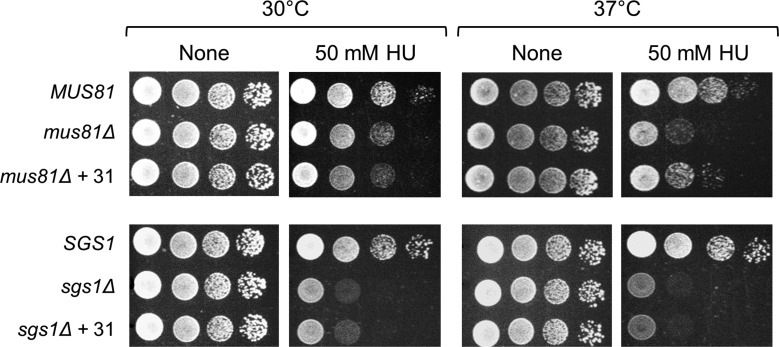
Transformation of plasmid number 31 can partially suppress the drug sensitivity of *mus81Δ* at 37 °C. NJY1777 strain was transformed with an empty vector pRS315 or vectors containing *MUS81* or screened plasmid number 31. To create *sgs1Δ* mutant cells, NJY1777 strain was transformed with pRS314 vector containing *MUS81* and transformants were grown in the presence of 5-FOA to remove *pJM500-URA3-SGS1* plasmid.

We questioned that the suppression of HU sensitivity by Flp1 overexpression in *sgs1Δmus81Δ* cells expressing Mus81_Δ100_ is associated with the loss of N-terminal region of Mus81 which is equivalent to loss of Mus81 function, or the loss of Sgs1, or a combination of these defects. Thus, using the plasmid number 31, we examined the ability of Flp1 overexpression in rescuing cellular defects of the *mus81Δ* and *sgs1Δ* mutants, respectively and found that Flp1 overexpression was unable to suppress the HU sensitivity of *sgs1Δ* (Figure 5). The transformation of plasmid number 31 could not suppress the *mus81Δ* mutant defect at temperature 30 °C, however, it succeeded to make the mutant partially resistant to HU treatment at 37 °C (Figure 5). These findings, together with the screening results, indicate that the suppression by Flp1 is related to the loss of Mus81 function, not Sgs1.

**Figure 6.**
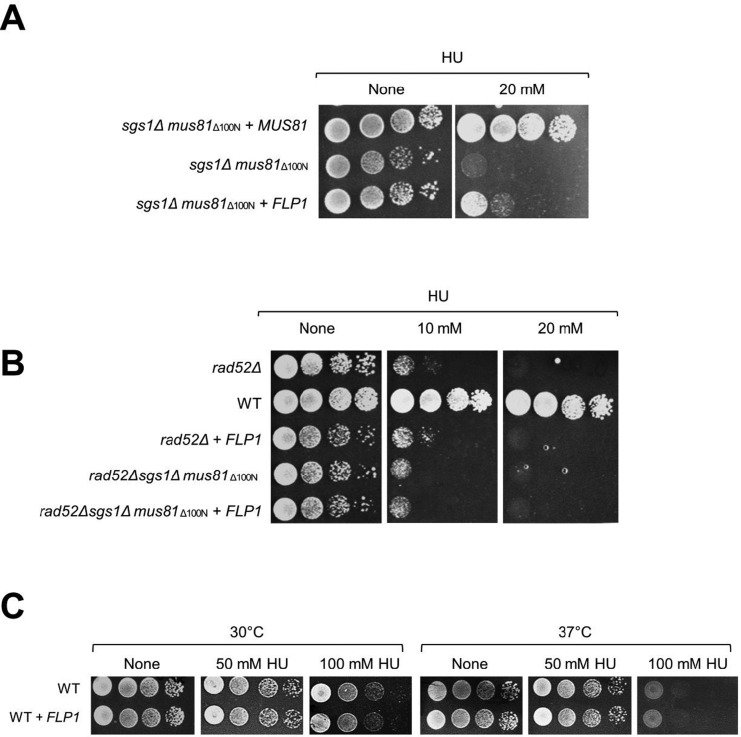
Suppression of drug sensitivity by Flp1 overexpression is specific for mus81 mutants. (A) Overexpression of Flp1 could partially rescue the HU-sensitivity of the *sgs1Δmus81_Δ100N_* cells. *FLP1* coding sequence was cloned into pRS315 plasmid and its expression was driven by *ADH1* promoter. Empty vector pRS315 or vector containing *MUS81* or *FLP1* was then transformed into the *sgs1Δmus81_Δ100N_* cells. (B) Overexpression of Flp1 could not suppress the HU-sensitivity of *rad52Δ* and *rad52Δsgs1Δmus81_Δ100N_* cells. (C) Overexpression of Flp1 could not rescue the HU-sensitivity of wild-type cells.

Next, *FLP1* gene was separately cloned into pRS315 vector and using drop dilution assay, we observed that the Flp1 overexpression could partially rescue the HU sensitivity of the *sgs1Δmus81_Δ100N_* mutant cells (Figure 6A). This result confirmed that Flp1 is a single-copy suppressor of the *sgs1Δmus81_Δ100N_* mutant. Besides, overexpression of Flp1 failed to suppress the HU sensitivity of *rad52Δ* and *rad52Δsgs1Δmus81_Δ100N_* cells (Figure 6B), indicating that Flp1 does not involve in the processing of intermediates at early stage of homologous recombination repair. Also, it was unable to rescue the wild type cells at high amount of HU treatment (Figure 6C), confirming the specific suppressive effect towards *mus81* mutants which lack the full function of Mus81-Mms4 *in vivo*. Flp1 catalyzes a site-specific recombination reaction via interacting with DNA sequences within its FRT sites, producing a single-stranded break on both DNA strands within the target sites that results in inversion of a segment of the 2-micron plasmid [38],[39]. Flp1 was determined to remain binding to the 3′ side of each break by an O-phosphotyrosyl residue [40]. Accordingly, Flp1 is a very promising suppressor because all of its activities are potentially required in homologous recombination repair pathway when Sgs1 is absent and Mus81 is partially dysfunctional.

## 4. Conclusions

In mitotic cells, the homologous recombination intermediates are preferentially processed by a ‘dissolution’ activity of the Sgs1-Top3-Rmi1 complex to merely generate non-crossover products. When cells are challenged with DNA damaging agents interfering with normal progression of replication forks or producing excessively double strand breaks, the joint work of Sgs1 and Mus81 could rapidly resolving the excess amount of DNA damages, despite of the risk of crossover product formation. Our previous study has shed light on the important role of Mus81 N-terminal region for the full function of Mus81 complex in the absence of Sgs1 during homologous recombination repair pathway [26]. Next, we raised a question for the alternative pathway that can rescue the cellular defect of *mus81* mutant isolated in the study. As described in Results section, we have succeeded to recover the *FLP1* gene on the 2-micron plasmid as the suppressor. The 2-micron circle independently reproduces itself with chromosome-like stability via the joined activity of a plasmid amplification system and a plasmid partitioning system. There has not been obvious evidence for the advantage or disadvantage of this plasmid existence to its host [33]. However, a very high copy numbers of the plasmid is harmful to the host, leading to cell cycle misregulation and cell lethality [34]. There is a fact that our strain cells should already harbor 2-micron plasmids but were failed to survive when treated with HU without being further transformed with an extra-plasmid containing *FLP1* sequence. This observation can be explained by regulation of the endogenous 2-micron plasmid number and gene expression. In details, three proteins Rep1, Rep2, and Faf1 which belong to the partitioning system control the copy number of plasmid by repressing expression of Flp1 protein. The amount of three repressors is in proportion to the 2-micron plasmid copy number, hence, expression of Flp1 is suppressed when the plasmid copy number is high, and if the plasmid copy number is small, Flp1 expression is induced. Therefore, expression of endogenous Flp1 is not relevant to the condition that the cell has to face. However, expression of Flp1 on an extra-plasmid transformed is segregated to the number and regulation of endogenous 2-micron system. The protein is considered as to be overexpressed and can be potentially beneficial when cells are challenged by DNA damaging agents.

We already prove that the lethality of *sgs1Δmus81_Δ120N_* was rescued by further deletion of Rad52, a key homologous recombination mediator in homologous recombination in budding yeast, indicating that the cellular defect related to dysfunction of Mus81 lacking N-terminal region was caused by the accumulation of unprocessed toxic recombination intermediates [26]. Therefore, the suppression of cellular defect of *sgs1Δmus81_Δ100N_* by Flp1 overexpression should be derived from the ability to reduce the late recombination intermediates accumulated. This notion is further supported by the result that overexpression of Flp1 was unable to rescue the cellular defect caused by the absence of Rad52 which functions upstream of Mus81 complex. The possibility that Flp1 can participate in processing toxic intermediates arising when cells are faced with DNA lesions was also supported by the observation that the overexpression of Flp1 can partially rescue the HU sensitivity of *mus81Δ* mutant at non-permissive temperature (37 °C). At 37 °C, a condition that the cells grow and divide rapidly, HU treatment induces quickly high accumulation of toxic intermediates that only Sgs1 activity could not cope with efficiently, representing by the highly drug-sensitive level of the cells (Figure 5). This excessive accumulation of recombination intermediates could lead to a condition that the cells may activate Flp1 function in mediating the removal of the toxic intermediates besides Sgs1, showing the partially resistant capability to drug (Figure 5).

Until now, the reason of 2-micron plasmid presence inside yeast cell has not been clearly defined, hence, identifying Flp1 as the suppressor of Mus81 partial dysfunction raises the possible explanation of advantage of readily containing this plasmid inside the cells as a backup system. As discussed above, we believe that Flp1 overexpression suppressed the cellular defects related to the loss of Mus81 function caused by the loss of N-terminal region via procession of toxic recombination intermediates. Although Flp1 tetramers might bind DNA in a configuration similar from recombination repair intermediates, Flp1 is a site-specific recombinase requiring a specific DNA sequence to induce DNA cleavage and recombination. It has not been completely clear that the Flp1 overexpression suppressor effect depends on its enzymatic activities, pointing to an involvement of DNA cleavage and recombination, or its potential function is perhaps merely structural. Therefore, Flp1 should be further investigated for its possible function in resolving homologous recombination intermediates generated when cells try to repair DNA damages.
